# miR-135b expression downregulates Ppm1e to activate AMPK signaling and protect osteoblastic cells from dexamethasone

**DOI:** 10.18632/oncotarget.12138

**Published:** 2016-09-20

**Authors:** Jian-Bo Fan, Jian-Wei Ruan, Wei Liu, Lun-Qing Zhu, Xin-Hui Zhu, Hong Yi, Sheng-Yu Cui, Jian-Ning Zhao, Zhi-Ming Cui

**Affiliations:** ^1^ The Department of Orthopaedics, The Second Affiliated Hospital of Nantong University, Nantong 226001, Jiangsu, PR China; ^2^ The Department of Orthopedics, The Second Affiliated Hospital of Soochow University, Suzhou 215000, China; ^3^ The Center of Diagnosis and Treatment for Childrens' Bone Disease, Childrens' Hospital Affiliated to Soochow University, Suzhou 215000, Jiangsu, PR China; ^4^ Department of Orthopaedics, Jinling Hospital, Nanjing Medical University, Nanjing 210008, Jiangsu, PR China

**Keywords:** dexamethasone (Dex), osteoblastic cells, microRNA-135b, AMP-activated protein kinase (AMPK), phosphatase 1E (Ppm1e)

## Abstract

Activation of AMP-activated protein kinase (AMPK) could potently protect osteoblasts/osteoblastic cells from dexamethasone (Dex). We aim to induce AMPK activation via microRNA (“miRNA”) downregulation of its phosphatase Ppm1e. We discovered that microRNA-135b (“miR-135b”) targets the 3' untranslated regions (UTRs) of Ppm1e. In human osteoblasticOB-6 cells and hFOB1.19 cells, forced-expression of miR-135b downregulated Ppm1e and activated AMPK signaling. miR-135b also protected osteoblastic cells from Dex. shRNA-induced knockdown of Ppm1e similarly activated AMPK and inhibited Dex-induced damages. Intriguingly, in the Ppm1e-silenced osteoblastic cells, miR-135b expression failed to offer further cytoprotection against Dex. Notably, AMPK knockdown (via shRNA) or dominant negative mutation abolished miR-135b-induced AMPK activation and cytoprotection against Dex. Molecularly, miR-135b, via activating AMPK, increased nicotinamide adenine dinucleotide phosphate (NADPH) activity and inhibited Dex-induced oxidative stress. At last, we found that miR-135b level was increased in human necrotic femoral head tissues, which was correlated with Ppm1e downregulation and AMPK activation. There results suggest that miR-135b expression downregulates Ppm1e to activate AMPK signaling, which protects osteoblastic cells from Dex.

## INTRODUCTION

Dexamethasone (Dex) and other glucocorticoids (GC) are often prescribed to patients with inflammatory and auto-immune diseases [1]. Yet, Dex will induce cytotoxic effect to osteoblasts, which may contribute to osteoporosis and osteonecrosis [2, 3]. Studies are focusing on exploring the pathological mechanisms of GC-induced osteoblast damages, and to develop possible intervention strategies [4–9]. It has also been our research focus [5, 7].

AMP-activated protein kinase (AMPK) is the master regulator of energy metabolism in mammalian cells [10]. Existing evidences have indicated that AMPK-regulated signalings are also important for cell survival, especially when cells are under certain stress conditions [11]. AMPK could activate cytoprotective autophagy to inhibit cell apoptosis [12, 13]. Meanwhile, AMPK may also exert an anti-oxidant activity through activating nicotinamide adenine dinucleotide phosphate (NADPH) and inhibiting ATP depletion [14].

Recent studies have investigated the potential functions of AMPK in osteoblasts/osteoblastic cells. Guo et al., showed that activation of AMPK by a novel AMPK activator (Compound 13 [15]) protected osteoblasts from Dex [16]. Meanwhile, A-769662, the known AMPK activator, could offer significant protection to osteoblasts against hydrogen dioxide (H_2_O_2_) [17]. On the other hand, inhibition of AMPK potentiated H_2_O_2_-induced osteoblast cell damages [18]. These results suggest that activation of AMPK likely exerts cytoprotective functions in osteoblasts/osteoblastic cells.

Phosphorylation on Thr172 of AMPK catalytic α subunit is vital for AMPK activation [13, 19]. Although kinase phosphorylation of this site has been extensively studied [20], the phosphatases that de-phosphorylate it are largely unknown until recently [21]. The initial data have demonstrated that protein phosphatase ce:sup>/ce:sup>/Mn2+-dependent (Ppm) 1e (Ppm1e) could be a primary AMPK kinase phosphatase [21]. Ppm1e, a PPM family member, was first identified as a Ca^2+^/calmodulin-dependent protein kinase (CaMK) phosphatase. It is activated in the presence of Mg^2+^ and/or Mn^2+^ [22, 23]. Ppm1e is insensitive to PPP family inhibitors (*i.e.* calyculin A and okadaic acid) [22, 23]. Studies have shown that Ppm1e can de-phosphorylate CaMKI, nuclear CaMKIV, and CaMKII [22, 23].

Based on the above discussion, we would propose that inhibition of Ppm1e may activate AMPK signaling and protect osteoblasts/osteoblastic cells from Dex. Through searching miRNA databases, we found that microRNA-135b (“miR-135b”) targets the 3' untranslated regions (UTRs) of Ppm1e. We showed that miR-135b expression downregulated Ppm1e to activate AMPK, which protects osteoblastic cells from Dex.

## RESULTS

### Forced expression of microRNA-135b downregulates Ppm1e but activates AMPK signaling in human osteoblastic cells

**Figure 1:**
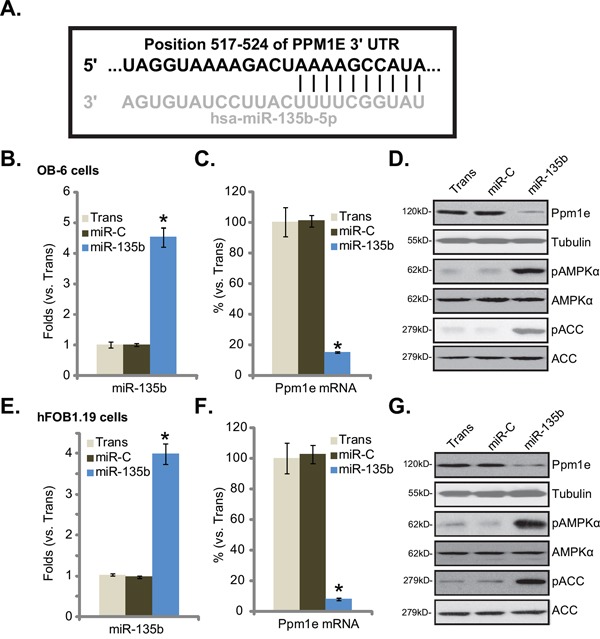
Forced expression of microRNA-135b downregulates Ppm1e but activates AMPK signaling in human osteoblastic cells **A.** microRNA-135b (“miR-135b”) targets the 3' untranslated regions (UTRs, position 517-524) of human Ppm1e (Figure 1A). Human osteoblastic OB-6 cells **B-D.** or hFOB1.19 cells **E-G.** were transfected with microRNA-135b (“miR-135b”) or non-sense control microRNA (“miR-C”), and stable cells were established. Expressions of miR-135b (B and E) and Ppm1e mRNA (C and F) were tested by quantitative real-time PCR (“qRT-PCR”) assay; Expression of listed proteins in these cells were tested by Western blot assay (D and G). Experiments in this figure were repeated four times, and similar results were obtained. “Trans” stands for transfection reagents only (B-G). *p<0.05 vs. group “miR-C” (B, C, E and F).

First, through searching multiple microRNA database, we found that microRNA-135b (“miR-135b”) targets the 3' untranslated regions (UTRs, Position 517-524) of human Ppm1e (Figure 1A). We next established a miR-135b expressing construct (see Methods) and stably transfected it into osteoblastic cells. qRT-PCR assay results in Figure 1B showed that miR-135b was indeed over-expressed in the stable OB-6 cells after transfection. Expression of miR-135b-5p was also significantly increased (Data not shown). On the other hand, Ppm1e mRNA (Figure 1C) and protein (Figure 1D) were both dramatically downregulated. Importantly, p-AMPKα (Thr-172) and p-acetyl-CoA carboxylase (p-ACC, Ser-79) levels were largely enhanced in the miR-135b-expresising OB-6 cells (Figure 1D), indicating AMPK signaling activation. We repeated those experiments also in osteoblastic hFOB1.19 cells. Similarly, stable hFOB1.19 cells expressing miR-135b (Figure 1E) showed depleted Ppm1e (Figure 1F and 1G) but increased AMPK activation (p-AMPKα/p-ACC, Figure 1G). Together, these results suggest that forced expression of miR-135b decreases Ppm1e, but activates AMPK signaling in cultured human osteoblastic cells.

### Forced expression of microRNA-135b protects osteoblastic cells from Dex

**Figure 2:**
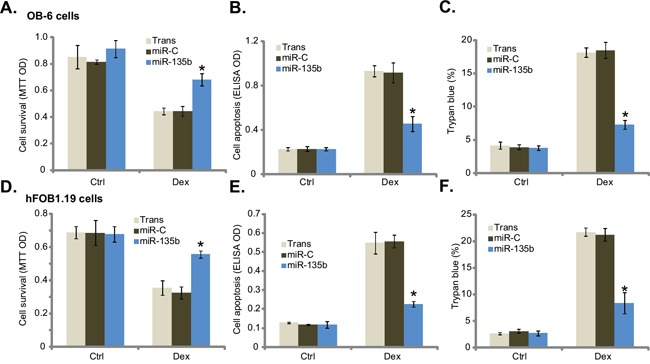
Forced expression of microRNA-135b protects osteoblastic cells from Dex Stable osteoblastic OB-6 cells **A-C.** or hFOB1.19 cells **D-F.** expressing microRNA-135b (“miR-135b”) or non-sense control microRNA (“miR-C”) were treated with or without Dex (1 μM) for 24 hours, cell viability (MTT assay, A and D), apoptosis (Histone DNA ELISA assay, B and E) and cell death (trypan blue assay, C and F) were tested. Experiments in this figure were repeated four times, and similar results were obtained. “Ctrl” stands for untreated control group. “Trans” stands for transfection reagents only. *p<0.05 vs. “miR-C” cells with Dex treatment.

Recent studies have indicated that activation of AMPK could protect osteoblastic cells [16–18]. The results above demonstrated that AMPK was activated in miR-135b-expressing osteoblastic cells. We therefore wanted to know if these cells were actually protected from Dex. OB-6 osteoblastic cells were treated with Dex, which induced viability inhibition (MTT OD reduction, Figure 2A), apoptosis activation (Histone DNA ELISA OD increase, Figure 2B) and cell death (trypan blue increase, Figure 2C). Remarkably, expression of miR-135b significantly attenuated above cytotoxic effects by Dex in OB-6 cells (Figure 2A-2C). In another words, OB-6 cells with miR-135b expression were resistant to Dex (Figure 2A-2C). We also repeated those experiments in hFOB1.19 cells, and similar anti-Dex effects by miR-135b were noticed (Figure 2D-2F). As compared to control cells, hFOB1.19 cells expressing miR-135b were protected from Dex, showing improved viability (Figure 2D), decreased cell apoptosis (Figure 2E) and cell death (Figure 2F). These results suggest that forced expression of miR-135b indeed protects osteoblastic cells from Dex.

### shRNA knockdown of Ppm1e activates AMPK and protects osteoblastic cells from Dex

**Figure 3:**
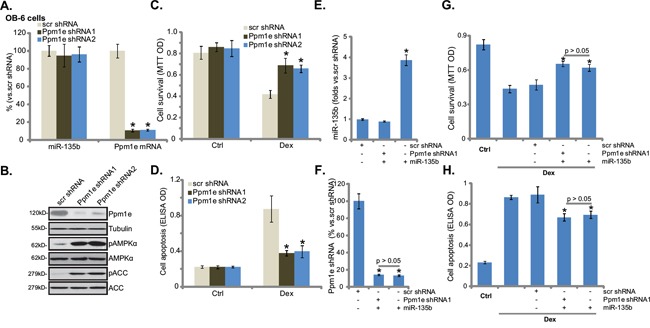
shRNA knockdown of Ppm1e activates AMPK and protects osteoblastic cells from Dex OB-6 cells were infected with lentiviral Ppm1e shRNA (“−1 or −2”) or non-sense control shRNA (“scr shRNA”), and stable cells were established. miR-135b and Ppm1e mRNA levels were tested (**A**, qRT-PCR assay), Ppm1e protein expression and AMPK activation (p-AMPK/p-ACC) were also tested (**B**, Western blot assay). Above cells were treated with or without Dex (1 μM) for 24 hours, cell viability (**C**, MTT assay) and apoptosis (**D**, Histone DNA ELISA assay) were shown. Ppm1e shRNA (“-1”)-expressing OB-6 cells were transfected with miR-135b expressing construct, expressions of miR-135b **E.** and Ppm1e mRNA **F.** were tested by qRT-PCR assay, these cells were also treated with or without Dex (1 μM) for 24 hours, cell viability **G.** and apoptosis **H.** were shown. “Ctrl” stands for untreated control group. Experiments in this figure were repeated four times, and similar results were obtained. *p<0.05 vs. “scr shRNA” cells (A, C-H).

We wanted to know if Ppm1e is the primary target of miR-135b in mediating its cytoprotective effect in osteoblastic cells. First, shRNA strategy was applied to stably knockdown Ppm1e. qRT-PCR results in Figure 3A and Western blot results in Figure 3B confirmed Ppm1e knockdown by the targeted shRNAs in OB-6 cells. Notably, two shRNAs (“1/2”) targeting non-overlapping sequences of human *Ppm1e* were applied, and each of them efficiently downregulated Ppm1e (Figure 3A and 3B). On the other hand, AMPK activation, evidenced by p-AMPK/p-ACC, was increased in Ppm1e-silneced OB-6 cells (Figure 3B). Significantly, Dex-induced OB-6 cell viability reduction (Figure 3C) and apoptosis activation (Figure 3D) were dramatically attenuated in Ppm1e-knockdown cells. The Ppm1e shRNA experiments were also repeated in hFOB1.19 cells, and similar results were obtained (Data not shown).Therefore, Ppm1e silence activates AMPK and protects osteoblastic cells from Dex.

We also expressed miR-135b in the Ppm1e-silenced OB-6 cells. qRT-PCR results in Figure 3E confirmed miR-135b over-expression in Ppm1e-shRNA (“-1”)-containing OB-6 cells. Ppm1e mRNA level was again decreased in these cells (Figure 3F). Significantly, expression of miR-135b failed to further offer cytoprotection against Dex in the Ppm1e-silenced cells (Figure 3G and 3H). These results suggest that Ppm1e is likely the primary target of miR-135b in mediating its cytoprotective effect in osteoblastic cells.

### AMPK knockdown or mutation abolishes miR-135b-induced cytoprotection in osteoblastic cells

**Figure 4:**
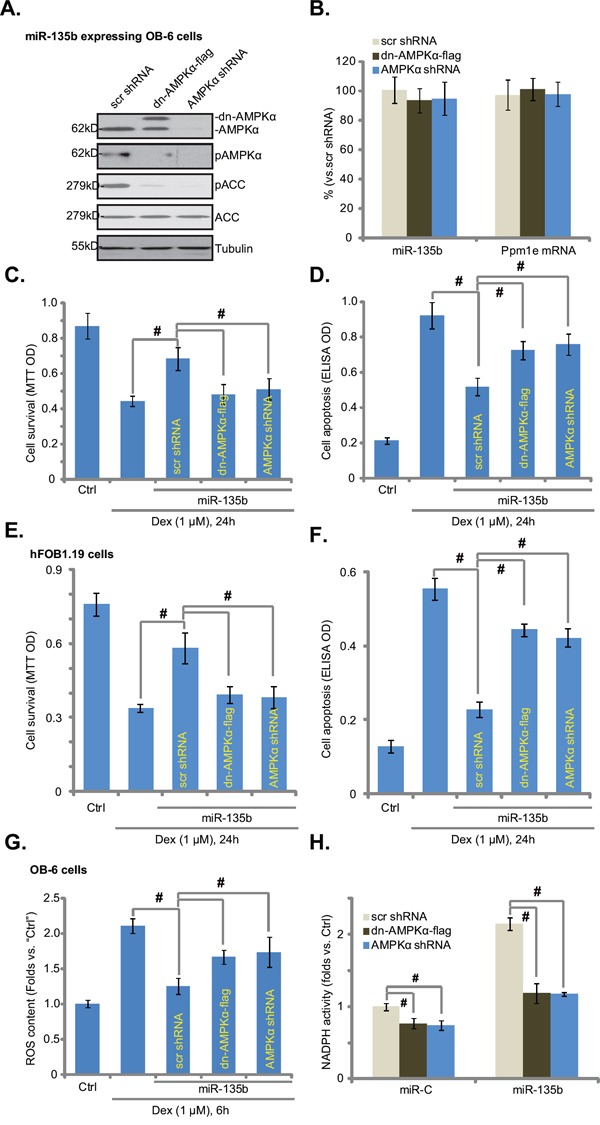
AMPK knockdown or mutation abolishes miR-135b-induced cytoprotection in osteoblastic cells miR-135b expressing OB-6/hFOB1.19 cells cells were constructed with AMPKα shRNA, dominant negative AMPKα (“dn-AMPKα-flag”, T172A) or the scramble control shRNA (“Scr shRNA”), expressions of listed proteins in these cells were tested by Western blots (**A**, for OB-6 cells); miR-135b and Ppm1e mRNA expressions were also tested (**B**, qRT-PCR assay, for OB-6 cells). Above cells were treated with or without Dex (1 μM) for indicated periods of time, cell viability (MTT assay, **C** and **E**), apoptosis intensity (Histone DNA ELISA assay, **D** and **F**) and ROS content (DCFH-DA fluorescent dye assay, **G**) were tested. NADPH activity in above cells was also shown **H.** Experiments in this figure were repeated four times, and similar results were obtained. “Ctrl” stands for untreated control group. ^#^*p*<0.05.

Next, we wanted to know if AMPK activation was the reason of miR-135b-meidated cytoprotection in osteoblastic cells. Genetic strategies were applied to inhibit AMPK activation. AMPKα shRNA or a dominant negative AMPKα (dn-AMPKα, T172A) was introduced to the miR-135b-expressing OB-6 cells. Western blot results in Figure 4A confirmed the phenotypes (AMPKα knockdown or mutation) of these cells. Expectably, miR-135b or Ppm1e mRNA expression was not changed in these cells (Figure 4B). Importantly, AMPK knockdown or dominant negative mutation almost blocked AMPK activation (p-AMPK/p-ACC) in miR-135b-expressing OB-6 cells (Figure 4A). Consequently, miR-135b-induced cytoprotection against Dex was almost nullified in OB-6 cells (Figure 4C and 4D). In another words, miR-135b was no longer cytoprotective in AMPK-depleted or AMPK-mutated cells. Similar in hFOB1.19 cells, AMPKα shRNA or dominant negative mutation largely attenuated miR-135b-induced cytoprotection against Dex (Figure 4E and 4F). Based on these results, we conclude that AMPK activation mediates miR-135b-induced cytoprotection against Dex in osteoblastic cells.

Recent studies have shown that AMPK activation inhibits Dex-induced ROS production, therefore protecting osteoblasts/osteoblastic cells [16]. We also noticed a significant ROS increase in Dex-treated OB-6 cells (Figure 4G). Such an effect by Dex was largely inhibited by expression of miR-135b (Figure 4G). Significantly, AMPK knockdown or dominant negative mutation almost completely abolished miR-135b-induced anti-oxidant ability (Figure 4G). It has been suggested that AMPK exerts anti-oxidant activity via activating NADPH [14, 16, 18, 24]. Here, we found that miR-135b-induced NADPH activity increase required AMPK activation (Figure 4H). AMPK shRNA knockdown or dominant negative mutation almost completely blocked NADPH activity increase by miR-135b (Figure 4H). The similar results were also obtained in hFOB1.19 cells (Data not shown). Thus, miR-135b activates AMPK-NADPH signaling to possibly inhibit Dex-induced oxidative stress and osteoblastic cell death.

### Upregulation of miR-135b in patients' osteonecrosis tissues

**Figure 5:**
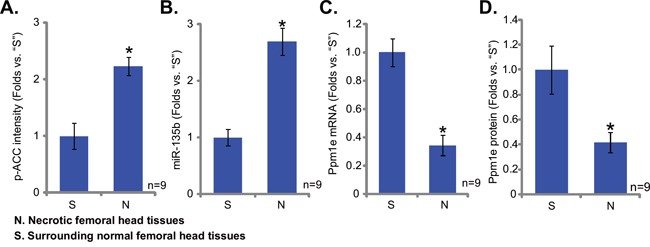
Upregulation of miR-135b in patients' osteonecrosis tissues Western blots analyzing the expression of listed proteins in surgery isolated femoral head tissues (both normal and necrotic) from GC-taking patients; ACC phosphorylation **A.** and Ppm1e protein (**D**, normalized to Tubulin) were quantified. Expressions of miR-135b **B.** and Ppm1e mRNA **C.** were also shown (qRT-PCR assay) in above tissues. *p<0.05 vs. “S” tissues.

A recent study by She *et al.,* showed that, as compared to the normal femoral head tissues, AMPK activation was increased in patients' necrotic femoral head tissues [18]. Here, we also observed AMPK activation (p-ACC intensity increase) in the necrotic femoral head tissues (*vs.* the surrounding normal femoral head tissues) (Figure 5A). Interestingly, in the necrotic tissues, the level of miR-135b was also increased (Figure 5B), and the Ppm1e (protein and mRNA) level was decreased (Figure 5C and 5D). These results indicate that miR-135b upregulation and Ppm1e depletion could be the reason of AMPK activation in patients' necrotic femoral head tissues.

## DISCUSSIONS AND CONCLUSIONS

Here we showed that miR-135b selectively targets the AMPK phosphatase Ppm1e [21], whose expression induced a profound AMPK activation in osteoblastic cells. Significantly, miR-135b expression or Ppm1e shRNA knockdown, which also activated AMPK signalings, significantly protected osteoblastic cells from Dex. Intriguingly, after Ppm1e was silenced, miR-135b was not able to further protect osteoblastic cells from Dex. Therefore, Ppm1e could be the primary target of miR-135b in mediating its cytoprotective effect. Additionally, we showed that AMPK knockdown or mutation abolished miR-135b-induced cytoprotection against Dex in osteoblastic cells, supporting a critical function of AMPK activation in miR-135b's actions in osteoblastic cells.

Dex treatment is shown to induce oxidative stress in osteoblasts/osteoblastic cells, which subsequently contributes to cell death and apoptosis [16, 25]. On the other hand, ROS suppression could inhibit Dex-induced osteoblastic cell damages [16]. Interestingly, recent studies have proposed potent anti-oxidant activity of AMPK [14, 16, 18, 26]. AMPK is shown to participate in maintaining NADPH content [14]. AMPK phosphorylates and inhibits ACC, thus decreasing NADPH consumption [14]. Meanwhile, AMPK activation was shown to induce NADPH synthesis by fatty-acid oxidation [14]. A very recent study by Guo et al., showed that compound 13 (C13), an α1-selective AMPK activator, activated NADPH signaling to inhibit Dex-induced ROS production, therefore protecting osteoblastic cells from Dex [16]. Here we showed that miR-135b expression increased NADPH activity and inhibited Dex-induced oxidative stress in osteoblastic cells, and such effects were almost reversed by AMPK knockdown or mutation. Therefore, we suggest that miR-135b activates AMPK to exert anti-oxidant activity and to protect osteoblastic cells from Dex.

Prolonged and/or overdose GC usage would cause secondary osteoporosis [27, 28] and/or osteonecrosis [29]. Reduced number of osteoblasts, suppressed osteoblastogenesis and increased osteoblast cell apoptosis were observed in the bones of the GC-taking patients [27, 28]. Here we found that miR-135b attenuated Dex-induced osteoblastic cell death and apoptosis. Significantly, miR-135b level was increased in human necrotic femoral head tissues, which was correlated with Ppm1e downregulation and AMPK activation. Thus, it will be interesting to test the possible effect of miR-135b against GC-induced osteoporosis and/or osteonecrosis *in vivo*. In summary, our preclinical results indicate that miR-135b expression downregulates Ppm1e to activate AMPK signaling and protect osteoblastic cells from Dex.

## MATERIALS AND METHODS

### Chemicals and reagents

Dex was purchased from Sigma (Shanghai, China). All cell culture reagents were obtained from Gibco (Shanghai, China). Antibodies of p-AMPKα1 (Thr172, #2531), AMPKα (#2532), acetyl-CoA Carboxylase (ACC, #3662), p-ACC (Ser79, #3661) and (β-) Tubulin (#2146) were purchased from were obtained from Cell Signaling Technology (Beverly, MA). Ppm1e antibody was purchased from Abnova (#PAB21197, Taibei, China).

### Cell culture

The OB-6 [2] and hFOB1.19 [30] human osteoblastic cells were purchased from the Cell Bank of Shanghai Institute of Biological Science (Shanghai, China), and were cultured as described [2, 30].

### Quantitative real-time polymerase chain reaction (qRT-PCR) assay

Total RNA was extracted with the SV total RNA purification system (Promega, Shanghai, China). RNA was then reverse-transcribed through the reverse transcriptase (Promega, Madison, WI, USA). cDNA derived from 1.0 μg of total RNA was amplified by quantitative real-time polymerase chain reaction (“qRT-PCR”). The SYBR Green PCR kit (Applied Biosystems) was then utilized to detect expression of listed mRNAs. *GAPDH* primers were described in our previous studies [31]. Human *Ppm1e* primers were described in previous studies [32]. PCR was performed in triplicate and was conducted using a Real-Time PCR Detection System (7500; ABI, Shanghai, China). mRNA expression was quantified using the ^ΔΔ^Ct method. *GAPDH* served as the internal control. For miRNA analysis, real-time PCR was performed using PrimeScript miRNA RT-PCR Kit (Takara, Tokyo, Japan) according to the manufacturer's protocols. The miR-135b primers were described early [33, 34]. All the primers and sequences were synthesized by Genepharm (Shanghai, China).

### Forced miR-135b expression

Pre-miR-135b (see its sequence in studies [33, 34]) was sub-cloned into pSuper-neo (OligoEngine, Seattle, WA) to generate miR-135b expression vector. Osteoblastic cells were seeded onto 6-well plates with 50-60% confluence. The miR-135b construct (0.10 μg/mL of each well) was transfected to the osteoblastic cells via Lipofectamine 2000 protocol (Invitrogen, Shanghai, China). After 24 hours, cells were subjected to neomycin (1.0 μg/mL) selection for 10-12 days. miR-135b expression in the resulting stable cells was tested by the qRT-PCR assay. Control cells were transfected with non-sense scramble microRNA-control (“miR-C”) (a gift from Dr. Lu's group [35]).

### Western blot assay

As described in our previous studies [5, 7], cell lysates were extracted via RIPA lysis buffer (Bio-sky, Nanjing, China). Protein concentration was determined, and aliquots of 30 μg lysates per sample were electro-transferred on 10-12% SDS-PAGE gel, following by transfer to PVDF membranes. The blots were then incubated with designated primary antibodies and appropriate secondary antibodies. The antigen-antibody binding was detected via enhanced chemiluminescence (ECL) reagents.

### Cell death detection

Osteoblastic cells were seeded onto 12-well plates. Following the indicated treatment, cells were trypsinized. Cell death percentage was determined by counting cells using a cytometer after addition of trypan blue, which stained the cytoplasm of dead cells. Cell death percentage (%) = the number of trypan blue stained cells/the number of total cells (×100%) [7].

### Cell viability assay

Cell viability was measured via the routine 3-[4,5-dimethylthylthiazol-2-yl]-2,5 diphenyltetrazolium bromide (MTT) assay described in our previous studies [5, 7].

### Apoptosis assay by enzyme-linked immunosorbent assay (ELISA)

As described [31], we applied the Histone-DNA ELISA Detection Kit (Roche, Palo Alto, CA) to quantify cell apoptosis following indicated treatments.

### shRNA knock and stable cell selection

The two lentiviral shRNAs against human *Ppm1e* were designed, synthesized and verified by Genepharm (Shanghai, China). The AMPKα shRNA was described in our previous study [31]. Osteoblastic cells were seeded onto 6-well plates with 50% of confluence. The lentiviral shRNA (10 μL/mL) were added directly to the cells for 24 hours. Afterwards, cells were further cultured in puromycin (1 μg/mL)-containing medium, until resistant colonies can be identified (2-3 weeks). The expression of target protein (AMPKα or Ppm1e) in stable cells was tested by Western blot assay. Same amount of scramble non-senesce lentiviral shRNA (Santa Cruz) was added to the control cells.

### AMPK dominant negative mutation

The dominant negative mutant of AMPKα (dn-AMPKα, T172A) construct was a gift from Dr. Lu's group at Nanjing Medical University [36]. Osteoblastic cells were seeded onto 6-well plates with 50% of confluence. dn-AMPKα cDNA (0.10 μg/mL) was transfected to osteoblastic cells via the Lipofectamine 2000 protocol [36], and stable cells were selected via neomycin (2 μg/mL, Sigma). Transfection efficiency was always verified via Western blot assay in the stable cells.

### NADPH activity assay

NADPH activity assay was described in our and other studies [18, 37]. Briefly, after treatment of cells, the lysates were incubated with NADP-cycling buffer plus glucose-6-phosphate dehydrogenase (G6PD, Sigma) at 60°C for 30 min [18]. Afterwards, glucose 6-phosphate (G6P, Sigma) was added to the mixture, and the change in absorbance at 570 nm was measured every 30 s for 4 min at 30°C. The concentration of NADP+ was calculated by subtracting [NADPH] from [total NADP]. NADPH activity was then calculated through NADPH/ NADP+ [37].

### Reactive oxygen species (ROS) measurement

ROS production was measured via a DCFH-DA fluorescent dye (Invitrogen). Briefly, following the applied treatment, cells were incubated with 1 μM of DCFH-DA at 37 °C for 30 min. Cells were then washed and analyzed for fluorescence using the flow cytometer (BD Biosciences, Shanghai, China). The ROS level in the treatment group was normalized to that of control group.

### Human tissue collection and analysis

Surgery-isolated fresh necrotic femoral head tissues and their surrounding normal femoral head tissues were collected from Dex-taking patients. Fresh tissue specimens were dissolved in tissue lysis buffer (BiYunTian Biotechnology Research Institute, Nantong, China) and were subjected to qRT-PCR assay and Western blot assay. The clinical examinations were approved by the institutional review board and ethics committee of all authors' institutions, and written informed consent was obtained from each patient. A total of 9 patients were included. All clinical investigations were conducted according to the principles expressed in the Declaration of Helsinki.

### Statistics

The data presented were mean ± standard deviation (SD). Statistical differences were analyzed by one-way ANOVA followed by multiple comparisons performed with post hoc Bonferroni test (SPSS version 18.0). Values of *p* < 0.05 were considered statistically significant. The significance of any differences between two groups was evaluated via the paired-samples *t* test (Excel, 2007) when appropriated.
